# Prevalence and risk factors of cardiovascular disease among people living with HIV in the Asia-Pacific region: a systematic review

**DOI:** 10.1186/s12889-023-15321-7

**Published:** 2023-03-13

**Authors:** Witchakorn Ruamtawee, Mathuros Tipayamongkholgul, Natnaree Aimyong, Weerawat Manosuthi

**Affiliations:** 1 Department of Epidemiology, Faculty of Public health, Mahidol University, 420/1 Ratchawithi Road, Ratchathewi, 10400 Bangkok, Thailand; 2 Department of Medicine, Department of Disease Control, Bamrasnaradura Infectious Diseases Institute, Ministry of Public Health, 11000 Nonthaburi, Thailand

**Keywords:** Risk factor, Cardiovascular diseases, HIV, Asia

## Abstract

**Background:**

Cardiovascular diseases (CVD) due to atherosclerosis have become one of the major causes of death among people living with HIV (PLHIV) since effective antiretroviral therapy (ART) has been available throughout the world. However, the epidemiologic evidence of this problem from the Asia-Pacific region remains unclear. We conducted a systematic review of the situation and risk factors for CVD among PLHIV in countries with the greatest impact of CVD attributable to HIV in the Asia-Pacific region.

**Methods:**

A systematic search in PubMed/MEDLINE, EMBASE, and the Cochrane Database of Systematic Reviews databases for articles published before 2019 was conducted. Publications reported situations and risk factors both traditional and HIV-specific for CVD among PLHIV in the region were included. Two reviewers working on duplicate and quality assessments, independently extracted data, and thematically analyzed the data.

**Results:**

Among PLHIV, the prevalence of subclinical CVD ranged from 10 to 28% and the incidence rate of clinical CVD ranged from 0.37 to 1.17 /100 person-years. Clinical CVD was frequently observed in the early era of the highly active antiretroviral therapy. A higher prevalence of subclinical CVD such as abnormal cIMT and carotid plaques was frequently observed in the PLHIV rather than in the nonHIV population and a high proportion of early onset of CVD was found among young PLHIV adults. The traditional risk factors for CVD such as hypertension, diabetes and smoking behavior were prevalent in both PLHIV and nonHIV populations ranging from 5 to 45%. HIV-specific risk factor, and lower CD4 presented almost twice the significantly increased risks for CVD while the synergistic interaction among traditional risk factors, i.e., diabetes mellitus, dyslipidemia and family history steeply increased the risk for CVD among PLHIV by almost 20 times.

**Conclusion:**

The limited existing data suggested the risk of early CVD among PLHIV. We identified the crucial gaps in HIV/CVD work from the Asia-Pacific region and recommended longer prospective studies with larger sample sizes or meta-analyses to better capture CVD risk and interactions of crucial risk factors of this vulnerable population in this region.

**Registration number:**

INPLASY202290108 (https://inplasy.com/inplasy-2022-9-0108/).

**Supplementary Information:**

The online version contains supplementary material available at 10.1186/s12889-023-15321-7.

## Background

The beneficial outcome of the highly active antiretroviral therapy (HAART) on people living with human immunodeficiency virus (PLHIV) is to increase quality of life and life expectancy [1]. The number of PLHIVs, aged over 50 years, has increased worldwide and is linked to an increased burden of noncommunicable diseases (NCDs). The double disease burden, HIV and NCDs in particular cardiovascular diseases (CVD), may cause a hectic burden on the economy of families and countries [2]. This situation requires global public health attention, particularly in low to middle income countries. From 1990 to 2015, the global burden of CVD attributable to HIV increased greater than three times from 0.74 million to 2.57 million disability-adjusted life-years [3]. A modeling study estimated that by the end of 2030 about 70% of PLHIV will be those aged older than 50 years, and 78% will have CVD [4].

A higher risk was observed of CVD among PLHIVs than that among HIV-negative individuals approximately 1.5 to 3 folds although the distribution of traditional risk factors for CVD among both groups did not differ [5–9]. Several recent studies suggested that underlying mechanisms of HIV-specific risk factors likely contributed to accelerated CVD in PLHIV, i.e., side effects of antiretroviral therapy (ART) and systemic chronic inflammation due to immune activation against HIV [5–10].

The Asia-Pacific region is the second region with a greater burden of HIV after sub-Saharan Africa, (5.8 and 25.7 million, respectively) [11]. The Asia-Pacific region has currently confronted the emerging challenge of CVD among PLHIV. A recent global burden of disease study revealed that the CVD population attributable to HIV was comparable with traditional risk factors [3]. Similarly, related studies suggested an increased incidence of CVD among PLHIV; however, most studies were conducted in high-income countries where epidemiologic evidence was unsuited for the Asia-Pacific region due to different socio-economic contexts [3, 7, 12–13]. To substantiate the situation of CVD and its risk factors among PLHIV remains indispensable for evidence-based public health in this low to middle-income region [3, 7, 9]. In this review, we addressed the knowable epidemiologic evidence of CVD among PLHIV in Asia-Pacific countries to provide existing scientific evidence to alert public health professionals in the region confronting the syndemic of HIV and CVD.

## Methods

### Search strategy

This systematic review of clinical and subclinical CVD among PLHIV in countries with the greatest impact of CVD attributable to HIV in the Asia-Pacific region, i.e., Thailand, Papua New Guinea, Bhutan, Cambodia, Myanmar, the Solomon Islands, Malaysia and Indonesia [3] was conducted according to the Preferred Reporting Items for Systematic Reviews and Meta-Analyses (PRISMA) guidelines [14] and reported following the Meta-analysis of Observational Research in Epidemiology guidelines [15]. This study has been registered in the INPLASY website with registration number INPLASY202290108 (https://inplasy.com/inplasy-2022-9-0108/).

The first author identified articles published in English through searching PubMed/MEDLINE, EMBASE, and the Cochrane Database of Systematic Reviews databases from any date to 31 December 2019 including Medical Subject Headings (MeSH) “Human immunodeficiency virus”, “People living with HIV”, “Cardiovascular”, “Cerebrovascular”, “Thailand”, “Cambodia”, “Myanmar”, “Bhutan”, “Papua New Guinea”, “the Solomon Islands”, “Malaysia”, or “Indonesia”. EndNote X8 (Clarivate Analytics, PA, USA) was used to collect, deduplicate, manage and review the searched articles. The detailed search strategy was provided in Additional file 1.

### Study eligibility

To identify the eligibility of each study, the procedure was performed in a stepwise manner. First, the titles and abstracts of the identified articles were screened for appropriateness by the first author (WR) consulting with the senior author (MT). All identified articles through database searching under the search strategy were included. We excluded non-English articles, before HAART implementation articles, conference abstracts, case reports/case series and randomized controlled trials. We also excluded studies not using cardiovascular disease as the outcome, not involving PLHIV and conducted among nonadult populations (ages < 18 years). Next, the selected article full texts were independently reviewed by two authors (WR and MT) to collect pertinent data in greater detail. Study selection disagreements were resolved by authors’ discussion. Finally, we extracted data for qualitative synthesis.

### Data extraction and quality assessment

Data were extracted on publication date, study area (country), study design, study period, sample size, age, CVD outcomes and measurements, incidence or prevalence of CVD outcomes among PLHIV and risk factors for CVD.

We used the Newcastle-Ottawa scale (N-O scale) to critically assess the quality of non-randomized studies [16]based on the study design, i.e., cohort, case-control and cross-sectional studies. The tool comprises three domains, i.e., participant selection, comparability and exposure/outcome assessment in the selected studies [17]. The N-O scale was then categorized in three levels following the standards of the Agency for Healthcare Research and Quality (AHRQ); good quality (> 3 stars in the selection domain, 1–2 stars in the comparability domain, 2–3 stars in the outcome/exposure domain); fair quality (2 stars in the selection domain and 1–2 stars in the comparability domain or 2–3 stars in the outcome/exposure domain); poor quality (< 1 star in selection domain or zero star in the comparability domain or < 1 star in the outcome/exposure domain) [17].

The data extraction and critical appraisal were conducted by the first author (WR). The second author (MT) independently checked and discussed all the results. In case of disagreement, the third author (NA) arbitrated. A detail of the quality assessment result is described in Additional File 2. We thematically analyzed and synthesized data from selected articles concerning the situation of CVD and risk factors which were classified as traditional risk factors and HIV-specific risk factors following Nou E et al. [18].

## Results

### Study selection and study characteristics

**Fig. 1:**
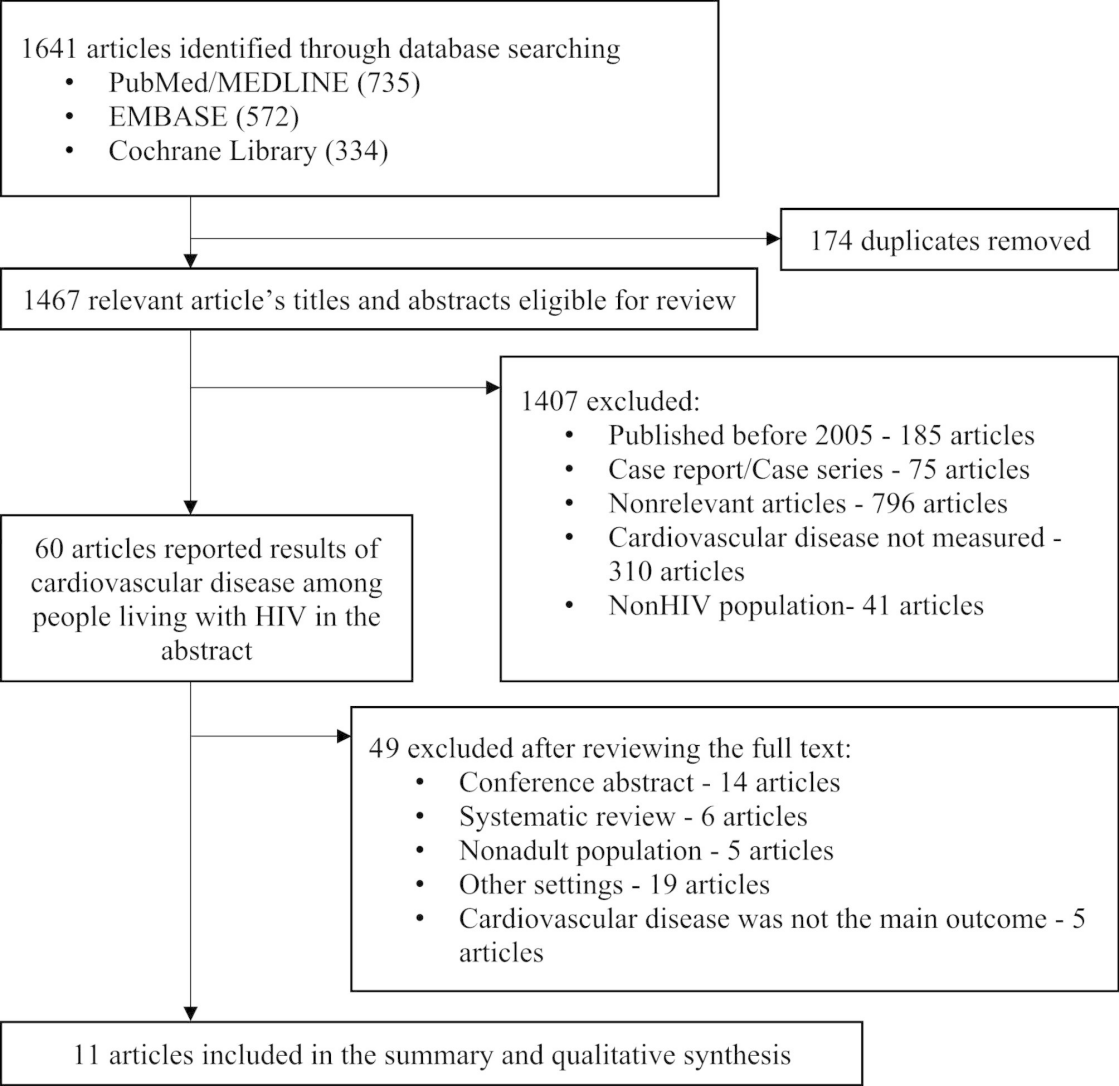
PRISMA Flow Diagram for Selection Eligible Study

A total of 1,641 articles were identified through the literature search. After removing all duplicates, titles and abstracts of 1,467 records were screened, and 1,407 articles were excluded. Of the 1,407 excluded articles, 185 were published in the pre-HAART era, 75 constituted case reports and case series, 796 revealed irrelevant study objectives, 310 lacked cardiovascular outcomes and 41 lacked PLHIV. We further searched for 60 full texts and then excluded 14 abstract conferences, 6 systematic reviews, 5 studies among child populations, 19 studies in other regions and 5 studies not measuring CVD outcomes. Finally, 11 eligible studies were included in the summary and qualitative synthesis (Fig. 1). Among 11 eligible studies, the study quality of 9 studies was good, and 2 studies were poor (Additional File 2).

The 11 studies, comprised 7 cross-sectional studies, 2 retrospective cohort studies, 1 prospective cohort study and 1 case-control study. Among 8 counties with the greatest impact of CVD attributable to HIV in the Asia-Pacific region, we found eligible publications from only 3, i.e., Indonesia, Malaysia and Thailand. The sample size of each study ranged from 50 to 1,813 subjects (Table 1).

**Table 1:** Published studies on CVD outcomes among PLHIV in Asia-Pacific counties with the greatest impact of CVD attributable to HIV status

Study (Country, year)	Study design	N =	Age (years)	Outcome measurements	Findings
Karim et al. 2017 [19] (Indonesia, 2013–2014)	Prospective cohort 1-year follow-up:	PLHIV: 67	Median: 31 years (range 19–48 years)	1. Carotid Intima-Media thickness (cIMT) 2. Diameter of carotid artery	1. Right cIMT among PLHIV (0.70 mm) was higher than uninfected (0.58 mm) after 12 months of ART initiation, *p* < 0.05. 2. Left cIMT among PLHIV (0.65 mm) was higher than that of uninfected (0.58 mm) after 12 months of ART initiation, *p* < 0.05. 3. No difference in the diameter of the right and left carotid artery after 12 months of ART initiation. 4. Traditional risk factors associated with cIMT and carotid artery diameter after 6 months of ART initiation, *p* < 0.05. 5. HIV-related factors associated with carotid artery diameter after 3 months of ART initiation, *p* < 0.05.
Subsai et al. 2006 [20] (Thailand, 2002–2004) *Poor quality	Retrospective Cohort 2 years follow-up:	PLHIV: 506	Mean: 36.8 ± 7.9	Hemorrhagic stroke or ischemic stroke	The incidence rate of hemorrhagic stroke:1.17/100 pys The incidence rate of ischemic stroke: 2.35/100 pys The incidence rate of stroke in the HAART era is higher than in the pre-HAART era.
Sitticharoenchai et al. 2019 [21] (Thailand, 2010–2015) *Good quality	Retrospective cohort 5 years follow-up: 2010 to 2015	PLHIV: 1,813	Median: 44 (range 38–50)	1. Coronary artery diseases 2. Ischemic stroke	1. Incidence rate of CVD: 3.75/1000 pys 2. Previous ischemic stroke and family history of CVD associated with CVD event, Adj OR 34.69 (95%CI 5.15-233.45) and Adj OR 6.89 (95%CI 2.57–18.48). 3. Interaction effect between diabetes mellitus and dyslipidemia on CVD: Adj OR 17.2 (95%CI 7.8–38.3) 4. Interaction effect between family history of CVD, diabetes mellitus and dyslipidemia on CVD: Adj OR 22.2 (95%CI 4.1-118.5). 5. High proportion of CVD among young HIV adult age < 55 years, 52.95%
Aurpibul et al. 2019 [22] (Thailand, 2015) *Good quality	Cross-sectional	PLHIV: 107 Non-HIV: 48	PLHIV Mean: 58.7 ± 6.5 Non-HIV Mean:59.7 ± 6.5	1. Subclinical atherosclerosis measured by Cardio-ankle vascular index (CAVI) 2. Peripheral artery disease (PAD) measured by Ankle- brachial index (ABI)	1. Prevalence of subclinical atherosclerosis (23% and 29%) and prevalence of PAD (6% and 8%) between PLHIV and NonHIV 2. DM associated with CAVI among PLHIV, Adj OR 1.54 (95%CI 1.01–2.35).
Putcharoen et al. 2019 [23] (Thailand, 2016–2017) *Good quality	Cross-sectional	PLHIV: 60 Non-HIV: 30	PLHIV Median: 54.9 (range 52–60) Non-HIV Median: 53 (range 50–60)	Carotid Intima-Media thickness (cIMT)	1. No difference in median overall cIMT between PLHIV (0.665 mm) and NonHIV (0.649 mm). 2. Of PLHIV, 10% was observed plague. 3. Male and hypertension associated with thicker cIMT among PLHIV, β = 0.041 (95%CI 0.001–0.081) and β = 0.047 (95%CI 0.003–0.092).
Siwamogsatham et al. 2019 [24] (Thailand, 2016–2017) *Good quality	Cross-sectional	PLHIV: 316	Median age: 54.4 (IQR 51.7–59.4)	Carotid Intima-Media thickness (cIMT)	1. Subclinical CVD 28.2% 2. Age positively associated with subclinical atherosclerosis, Adj OR 1.06 (95%CI 1.003–1.12). 3. CD4 count (< 200 cells/mm^3^) associated with subclinical atherosclerosis, Adj OR 1.80 (95%CI 1.02–3.18).
Utama et al. 2019 [25] (Indonesia, 2017) *Good quality	Cross-sectional	PLHIV: 50	Mean: 30.60 ± 5.58	Carotid Intima-Media thickness (cIMT)	1. Older age increases the diameter of cIMT at β 0.012 (95%CI 0.002–0.022). 2. CD4/CD8 ratio increases the diameter of cIMT at β= -0.791 (95%CI -0.99 to -0.592).
Rajasuriar et al. 2015 [26] (Malaysia, NA) *Good quality	Cross-sectional	PLHIV: 84	Median: 41 (IQR 36–46)	Carotid Intima-Media thickness (cIMT)	Prevalence of subclinical atherosclerosis: 27.4%
Aurpibul et al. 2019 [27] (Thailand, 2015) *Good quality	Cross-sectional	PLHIV: 362 Non-HIV^−^: 362	PLHIV: Mean 57.8 ± 5.6 Non-HIV: Mean 58.1 ± 5.7	Peripheral artery disease (PAD) measured by ABI	1. Prevalence of PAD among PLHIV (5%) and nonHIV (7%) did not differ. 2. Prevalence of abnormal ABI among PLHIV (20%) were lower than that of uninfected(27%), *p*0.03. 3. Female sex and underweight associated with abnormal ABI among PLHIV, Adj OR 2.09 (95%CI 1.20–3.67) and Adj OR 1.73 (95%CI 1.02–2.95).
Nakaranurack, Manosuthi. 2018 [28] (Thailand, 2011) *Good quality	Cross-sectional	PLHIV: 874	45.5 ± 8.3	CVD	Prevalence of CVD among PLHIV: 1.3%
Lee et al. 2012 [29] (Thailand, 2009–2010) *Good quality	Case-Control	PLHIV with stroke: 37 PLHIV without stroke: 74	PLHIV with stroke: 50.5 ± 11.1 PLHIV without stroke: 50.4 ± 13.4	Stroke (cerebral infarction and intracerebral hemorrhage)	Tuberculous meningitis (Adj OR 11.9; 95%CI 1.2-117.2) and smoking (Adj OR 6.9; 95%CI 2.3–21.2) are associated with stroke among PLHIV.

### CVD among PLHIV in the Asia-Pacific Region

Early detection of CVD was reported in 11 studies, 7 studies identified subclinical CVD diagnosed by CAVI [22], cIMT [19, 23–26], and ABI [19, 22, 27] and another 4 studies identified clinical CVD, i.e., stroke [20, 21, 28, 29] and atherosclerosis [21, 28].

Subclinical CVD, measured using ABI and cIMT reported in seven studies, ranged from 10 to 28% [19, 23–27]. A lower prevalence of subclinical CVD was reported in a study in a younger PLHIV population [25] than that in other studies [19, 20-24, 26, 27]. The prevalence of subclinical CVD such as atherosclerosis was higher in PLHIV than in nonHIV populations [19][23].

Clinical CVD, stroke and coronary artery diseases were reported in two cohort studies ranging from 0.37 to 1.17 /100 person-years [20–21] and the prevalence of 1.3% from a cross-sectional study [28] among PLHIV in Thailand. However, the incidence of ischemic stroke (2.35/100 person-years) was higher than hemorrhagic stroke (1.17/100 person-years) [20]. Clinical CVD was frequently observed in the early highly active ART era, and a higher incidence was observed in longer follow-up time.

### Risk factors for CVD among PLHIV

The characteristics of CVD risk factor data among PLHIV collected by selected studies were classified in two groups: traditional risk factors and HIV-specific risk factors for CVD including adverse effects of antiretroviral therapy and factors related to systemic immune activation and HIV status [22].

### Traditional risk factors for CVD among PLHIV

The traditional risk factors for CVD among PLHIV in the selected studies were frequently assessed. Moreover, these risk factors were prevalent in both PLHIV and nonHIV populations. Hypertension presented at 13 to 45%, diabetes mellitus was 5 to 24% [21, 24, 26, 27–28] and smoking was 13 to 45% [25–29]. Although the prevalence of traditional risk factors between the PLHIV and the nonHIV populations did not conclusively differ [21-23, 26], DM, dyslipidemia and family history presented synergistic effects on CVD risks reported in the PLHIV cohort.

### HIV-specific risk factors for CVD

Although several selected studies intended to identify associations between HIV-specific risk factors and CVD among PLHIV [21–26, 28, 30], only 3 of 11 studies demonstrated the association of HIV-specific risk factors and subclinical or clinical CVD status among PLHIV in this region [24]. Poor immune system (CD4 cell count < 200 or CD8/CD4 ratio < 1) increased the risk of subclinical CVD [24, 25]. A cross-sectional study in Thailand reported the association between low nadir CD4 counts (< 200 cells/mm^3^) and carotid artery stenosis (abnormal cIMT > 0.9 mm) and/or presence of carotid plaques (adj OR 1.80; 95%CI 1.02–3.18) [24] similar to a study in Indonesia (β= -0.791) [25]. One cohort study reported a relationship between the duration of ART exposure with abnormal cIMT [19]. However, other HIV-specific risk factors such as antiretroviral therapies, duration of antiretroviral therapies exposure, statin use, fibrosis-4 index and high sensitivity c-reactive protein did not present any association [24]. Although clinical CVD prevalence between nonHIV and PLHIV did not significantly differ in both populations aged above 55 years, the onset of subclinical CVD such as abnormal cIMT and carotid plaques was earlier among the PLHIV than among nonHIV [19, 22-24].

## Discussion

This systematic review revealed a higher prevalence of subclinical and clinical CVD among PLHIV than that in the nonHIV population, a higher risk of subclinical CVD among poorer immune PLHIV and synergistic interaction between diabetes mellitus, dyslipidemia and family history on CVD risk among PLHIV. Although this systematic review underscored the significant risk of CVD among PLHIV in the Asia-Pacific region, the limitation in existing data remains. First, published data included small sample sizes which were less likely to detect the statistical association. Additionally, cohort studies did not have a longer follow-up time and were less likely to capture clinical CVD risks. NonHIV populations in three cross-sectional studies were not tested for HIV so the nonHIV populations likely mixed with populations with and without HIV. Those limitations can lead to underestimating the CVD risk; therefore, the CVD risk among PLHIV in this study can be used to raise attention from public health professionals in this region. Additionally, most studies used a cross-sectional design which did not capture the risk of CVD over time, and our study includes only English publications to prevent the challenges of accurate translation. This study did not meta-analyze the pooled effect of CVD risk factors because HIV/CVD studies are very few and likely inadequate for statistical analysis.

Obviously, the prevalence of subclinical atherosclerosis among PLHIV are the same as that in nonHIV populations in those study populations aged above 50 years [22–23, 26]. This situation reflected the double burdens of diseases in this region. The PLHIV regularly visit hospitals and attend health education sessions to improve healthy lifestyles to reduce the risk of NCDs [22, 33]. However, a prospective cohort study reported a higher prevalence of subclinical CVD compared with nonHIV populations which was consistent with data from other parts of the world [3, 5, 8–9, 35]. The higher CVD risk among PLHIV is related to multifactorial factors, both traditional and HIV-related risk factors for CVD [5–9].

The associations between the major traditional risk factors and CVD among PLHIV found in our review were consistent with results from other related reviews [6–7, 36]. These factors including diabetes, hypertension, dyslipidemia and smoking served major roles to increase oxidative stress in the cardiovascular system and led to chronic systemic inflammation, endothelial dysfunction, atherosclerosis progression and direct effects on cardiac performance through abnormal hormones or cytokines secretions. [36–42] Identifying extremely high risks of CVD among PLHIVs with DM, dyslipidemia and family members with CVD remains crucial.

The existing data were only from cross-sectional studies which cannot assess all potential exposures and CVD events over time. the prospective ascertainment for CVD among PLHIV and potential exposures will provide crucial information to identify future optimal interventions in this region. Although the association between HIV-specific risk factors and CVD has been reported in other studies [7, 42], HIV management may differ across regions especially between high and low to middle income countries. The study of HIV-specific risk factors for CVD and synergistic effects between traditional and HIV-specific risk factors should be conducted in this region.

## Conclusion

The limited existing data suggested the risk of early CVD among PLHIV. Extreme CVD risk among PLHIV with DM, dyslipidemia, and family history should highlight the need of NCDs intensive prevention program. We identified the crucial gaps in HIV/CVD work from the Asia-Pacific Region and recommended a further prospective study with large sample size and longer follow-up time or conducting a meta-analysis to better capture CVD risk, and interaction between HIV-related and traditional risk factors in this vulnerable population in this region.

## Electronic supplementary material

Below is the link to the electronic supplementary material.

Supplementary Material 1

Supplementary Material 2

## Data Availability

The original articles included in this systematic review are publicly available. The complete search strategy is available in Additional file 1.

## Abbreviations

ABI: Ankle-Brachial Index
AHRQ: The Agency for Healthcare Research and Quality Standard
ART: Antiretroviral Therapy
CAVI: Cardio-Ankle Vascular Index
cIMT: Carotid Intima-Media Thickness
CVD: Cardiovascular Diseases
HIV: Human Immunodeficiency Virus
N-O scale: Newcastle-Ottawa scale
PLHIV: People Living with HIV

## References

[1] Burgoyne RW, Tan DHS (2008). Prolongation and quality of life for HIV-infected adults treated with highly active antiretroviral therapy (HAART): a balancing act. J Antimicrob Chemother.

[2] Liao C, Yang C, Chen P, Ou H, Toh HS, Chen Z, Ko N (2018). Direct medical costs associated with cardiovascular diseases among the HIV-infected patients in Taiwan: a population-based study. Value in Health.

[3] Shah ASV, Stelzle D, Lee KK, Beck EJ, Alam S, Clifford S (2018). Global Burden of Atherosclerotic Cardiovascular Disease in People living with HIV. Circulation.

[4] Smit M, Brinkman K, Geerlings S, Smit C, Thyagarajan K, Sighem A (2015). Future challenges for clinical care of an ageing population infected with HIV: a modelling study. Lancet Infect Dis.

[5] Kearns A, Gordon J, Burdo TH, Qin X (2017). HIV-1-Associated atherosclerosis: unraveling the Missing Link. J Am Coll Cardiol.

[6] Nou E, Lo J, Hadigan C, Grinspoon SK (2016). Pathophysiology and management of cardiovascular disease in patients with HIV. Lancet Diabetes Endocrinol.

[7] Vachiat A, McCutcheon K, Tsabedze N, Zachariah D, Manga P (2017). HIV and Ischemic Heart Disease. J Am Coll Cardiol.

[8] Feinstein MJ, Hsue PY, Benjamin LA, Bloomfield GS, Currier JS, Freiberg MS et al. Characteristics, Prevention, and Management of Cardiovascular Disease in People Living With HIV: a Scientific Statement From the American Heart Association.Circulation. 2019:CIR0000000000000695. 10.1161/CIR.0000000000000695 PMC7993364 31154814

[9] Hsue PY, Waters DD (2019). HIV infection and coronary heart disease: mechanisms and management. Nat Rev Cardiol.

[10] Ballocca F, D’Ascenzo F, Gili S, Grosso Marra W, Gaita F (2017). Cardiovascular disease in patients with HIV. Trends Cardiovasc Med.

[11] Joint United Nations Programme on HIV/AIDS. UNAIDS Data. 2019 Available at: http://rstesa.unaids.org/publications/global-publications/2019/item/208-unaids-data-2019. Accessed on April 6, 2022.

[12] Patel P, Rose CE, Collins PY, Nuche-Berenguer B, Sahasrabuddhe VV, Peprah E (2018). Noncommunicable diseases among HIV-infected persons in low-income and middle-income countries: a systematic review and meta-analysis. AIDS.

[13] So-Armah K, Freiberg MS (2018). HIV and Cardiovascular Disease: update on clinical events, special populations, and novel biomarkers. Curr HIV/AIDS Rep.

[14] Liberati A, Altman DG, Tetzlaff J, Mulrow C, Gotzsche PC, Ioannidis JP (2009). The PRISMA statement for reporting systematic reviews and meta-analyses of studies that evaluate healthcare interventions: explanation and elaboration. BMJ.

[15] Stroup DF, Berlin JA, Morton SC (2000). Meta-analysis of Observational Studies in Epidemiology: a proposal for reporting. JAMA.

[16] Higgins JPT, Green S, editors, editors. Cochrane Handbook for Systematic Reviews of Interventions Version 5.1.0 [updated March 2011]. The Cochrane Collaboration, 2011. Available from www.handbook.cochrane.org.

[17] Landman AJEMC, Don EE, Vissers G, Ket HCJ, Oudijk MA, de Groot CJM et al. Modified Newcastle Ottawa quality assessment scale and AHRQ standards. [Internet]. PLOS ONE; 2022 [cited 2022Sep13]. Available from: https://plos.figshare.com/articles/journal_contribution/Modified_Newcastle_Ottawa_quality_assessment_scale_and_AHRQ_standards_/19965756/1

[18] Nou E, Lo J, Hadigan C, Grinspoon SK. Pathophysiology and management of cardiovascular disease in patients with HIV. Lancet Diabetes Endocrinol. 2016 Jul;4(7):598–610. 10.1016/S2213-8587(15)00388-5. 10.1016/S2213-8587(15)00388-5 PMC4921313 26873066

[19] Karim B, Wijaya IP, Rahmaniyah R, Ariyanto I, Waters S, Estiasari R et al. Factors affecting affect cardiovascular health in Indonesian HIV patients beginning ART.AIDS research and therapy. 2017; 14(1). 10.1186/s12981-017-0180-9 PMC5580224 28859681

[20] Subsai K, Kanoksri S, Siwaporn C, Helen L, Kanokporn O, Wantana P (2006). Neurological complications in AIDS patients receiving HAART: a 2-year retrospective study. Eur J Neurol.

[21] Sitticharoenchai P, Putcharoen O, Buddhari W (2019). Incidence and risk factors of cardiovascular diseases among HIV patients in Thailand. J Med Assoc Thai.

[22] Aurpibul L, Srithanaviboonchai K, Rerkasem K, Tangmunkongvorakul A, Sitthi W, Musumari PM (2019). Prevalence of subclinical atherosclerosis and risk of atherosclerotic Cardiovascular Disease in older adults living with HIV. AIDS Res Hum Retroviruses.

[23] Putcharoen O, Pleumkanitkul S, Chutinet A, Vongsayan P, Samajarn J, Sophonphan J (2019). Comparable carotid intima-media thickness among long-term virologically suppressed individuals with HIV and those without HIV in Thailand. J virus eradication.

[24] Siwamogsatham S, Chutinet A, Vongsayan P, Samajarn J, Putcharoen O, Aponpong T (2019). Low CD4 cell counts are Associated with Carotid Plaque and Intima-Media thickness in Virologically suppressed HIV-Infected Asians older than 50 years. AIDS Res Hum Retroviruses.

[25] Utama S, Patriawan P, Dewi A (2019). Correlation of CD4/CD8 ratio with carotid intima-media layer thickness in HIV/AIDS patients at Sanglah General Hospital, Bali, Indonesia. Open access Macedonian journal of medical sciences.

[26] Rajasuriar R, Kong YY, Nadarajah R, Abdullah NK, Spelman T, Yuhana MY (2015). The CD14 C-260T single nucleotide polymorphism (SNP) modulates monocyte/macrophage activation in treated HIV-infected individuals. J translational Med.

[27] Aurpibul L, Sugandhavesa P, Srithanaviboonchai K, Sitthi W, Tangmunkongvorakul A, Chariyalertsak C (2019). Peripheral artery disease in HIV-infected older adults on antiretroviral treatment in Thailand. HIV Med.

[28] Nakaranurack C, Manosuthi W. Prevalence of Non-AIDS Comorbidities and Factors Associated with Metabolic Complications among HIV-infected Patients at a Thai Referral Hospital.Journal of the International Association of Providers of AIDS Care. 2018; 17. 10.1177/2325957417752256 PMC6748460 29357771

[29] Lee B, Anekthananon T, Poungvarin N, Nilanont Y (2012). Etiology and risk factors of stroke in HIV-infected patients in Siriraj Hospital: a case-control study. J Med Association Thailand = Chotmaihet thangphaet.

[30] Putcharoen O, Wattanachanya L, Sophonphan J, Siwamogsatham S, Sapsirisavat V, Gatechompol S (2017). New-onset diabetes in HIV-treated adults: predictors, long-term renal and cardiovascular outcomes. AIDS.

[31] Peonim V, Sujirachato K, Srisont S, Udnoon J (2012). Pathology of HIV seropositive: forensic autopsy study in a tertiary care hospital, Bangkok, Thailand. J Med Association Thailand = Chotmaihet thangphaet.

[32] Wongcharoen W, Suaklin S, Tantisirivit N, Phrommintikul A, Chattipakorn N (2014). QT dispersion in HIV-infected patients receiving combined antiretroviral therapy. Annals of noninvasive electrocardiology: the official journal of the International Society for Holter and Noninvasive Electrocardiology Inc.

[33] Wongcharoen W, Khienprasit K, Phrommintikul A, Sukonthasarn A, Chattipakorn N (2013). Heart rate variability and heart rate turbulence in HIV-infected patients receiving combination antiretroviral therapy. Annals of noninvasive electrocardiology: the official journal of the International Society for Holter and Noninvasive Electrocardiology Inc.

[34] Hsue PY, Waters DD (2018). Time to recognize HIV infection as a Major Cardiovascular Risk factor. Circulation.

[35] Ghosn J, Taiwo B, Seedat S, Autran B, Katlama C, Hiv (2018). Lancet.

[36] Ballocca F, Gili S, D’Ascenzo F, Marra WG, Cannillo M, Calcagno A (2016). HIV infection and primary Prevention of Cardiovascular Disease: lights and Shadows in the HAART era. Prog Cardiovasc Dis.

[37] Low Wang CC, Hess CN, Hiatt WR, Goldfine AB (2016). Clinical update: Cardiovascular Disease in Diabetes Mellitus: atherosclerotic Cardiovascular Disease and Heart failure in type 2 diabetes Mellitus - Mechanisms, Management, and clinical considerations. Circulation.

[38] Petrie JR, Guzik TJ, Touyz RM (2018). Diabetes, hypertension, and Cardiovascular Disease: clinical insights and vascular mechanisms. Can J Cardiol.

[39] Roy A, Rawal I, Jabbour S, Prabhakaran D et al. Tobacco and Cardiovascular Disease: a Summary of Evidence. In: rd, Prabhakaran D, Anand S, Gaziano TA, Mbanya JC, Wu Y, editors. Cardiovascular, Respiratory, and Related Disorders. Washington (DC) 2017. 30212086

[40] Lavie CJ, McAuley PA, Church TS, Milani RV, Blair SN (2014). Obesity and cardiovascular diseases: implications regarding fitness, fatness, and severity in the obesity paradox. J Am Coll Cardiol.

[41] Hemkens LG, Bucher HC (2014). HIV infection and cardiovascular disease. Eur heart J.

[42] Zanni MV, Schouten J, Grinspoon SK, Reiss P (2014). Risk of coronary heart disease in patients with HIV infection. Nat Rev Cardiol.

