# Supplementary material for: Prevalence and risk factors of cardiovascular disease among people living with HIV in the Asia-Pacific region: a systematic review
Source: BMC Public Health. 2023 Mar 13;23:477. doi: 10.1186/s12889-023-15321-7 (PMC10009940; doi:10.1186/s12889-023-15321-7)
Supplement: Supplementary file 1 — Supplementary Material 1 [file 12889_2023_15321_MOESM1_ESM.docx]

**Additional File 1**

**Prevalence and Risk Factors of Cardiovascular Disease among People Living with HIV in the Asia-Pacific Region: a systematic review**

**Search strategies**

*PubMed/MEDLINE - [MeSH Terms] OR [All Fields] - All times*

*735 articles*

(("hiv"[MeSH Terms] OR "hiv"[All Fields]) OR ("hiv"[MeSH Terms] OR "hiv"[All Fields] OR ("human"[All Fields] AND "immunodeficiency"[All Fields] AND "virus"[All Fields]) OR "human immunodeficiency virus"[All Fields]) OR (("humans"[MeSH Terms] OR "humans"[All Fields] OR "human"[All Fields]) AND ("immunologic deficiency syndromes"[MeSH Terms] OR ("immunologic"[All Fields] AND "deficiency"[All Fields] AND "syndromes"[All Fields]) OR "immunologic deficiency syndromes"[All Fields] OR ("immune"[All Fields] AND "deficiency"[All Fields]) OR "immune deficiency"[All Fields]) AND ("viruses"[MeSH Terms] OR "viruses"[All Fields] OR "virus"[All Fields])) OR ("acquired immunodeficiency syndrome"[MeSH Terms] OR ("acquired"[All Fields] AND "immunodeficiency"[All Fields] AND "syndrome"[All Fields]) OR "acquired immunodeficiency syndrome"[All Fields] OR "aids"[All Fields]) OR ("acquired immunodeficiency syndrome"[MeSH Terms] OR ("acquired"[All Fields] AND "immunodeficiency"[All Fields] AND "syndrome"[All Fields]) OR "acquired immunodeficiency syndrome"[All Fields]) OR ("acquired immunodeficiency syndrome"[MeSH Terms] OR ("acquired"[All Fields] AND "immunodeficiency"[All Fields] AND "syndrome"[All Fields]) OR "acquired immunodeficiency syndrome"[All Fields] OR ("acquired"[All Fields] AND "immune"[All Fields] AND "deficiency"[All Fields] AND "syndrome"[All Fields]) OR "acquired immune deficiency syndrome"[All Fields]) OR ("hiv seropositivity"[MeSH Terms] OR ("hiv"[All Fields] AND "seropositivity"[All Fields]) OR "hiv seropositivity"[All Fields] OR ("hiv"[All Fields] AND "positive"[All Fields]) OR "hiv positive"[All Fields]) OR (("hiv seropositivity"[MeSH Terms] OR ("hiv"[All Fields] AND "seropositivity"[All Fields]) OR "hiv seropositivity"[All Fields] OR ("hiv"[All Fields] AND "positive"[All Fields]) OR "hiv positive"[All Fields]) AND ("patients"[MeSH Terms] OR "patients"[All Fields])) OR ("hiv infections"[MeSH Terms] OR ("hiv"[All Fields] AND "infections"[All Fields]) OR "hiv infections"[All Fields] OR ("human"[All Fields] AND "immunodeficiency"[All Fields] AND "virus"[All Fields] AND "infection"[All Fields]) OR "human immunodeficiency virus infection"[All Fields]) OR ("hiv-1"[MeSH Terms] OR "hiv-1"[All Fields] OR "hiv 1"[All Fields]) OR ("hiv-2"[MeSH Terms] OR "hiv-2"[All Fields] OR "hiv 2"[All Fields]) OR (("humans"[MeSH Terms] OR "humans"[All Fields] OR "human"[All Fields]) AND ("immunologic deficiency syndromes"[MeSH Terms] OR ("immunologic"[All Fields] AND "deficiency"[All Fields] AND "syndromes"[All Fields]) OR "immunologic deficiency syndromes"[All Fields] OR ("immuno"[All Fields] AND "deficiency"[All Fields]) OR "immuno deficiency"[All Fields]) AND ("viruses"[MeSH Terms] OR "viruses"[All Fields] OR "virus"[All Fields])) OR ("hiv infections"[MeSH Terms] OR ("hiv"[All Fields] AND "infections"[All Fields]) OR "hiv infections"[All Fields]) OR HIV-infected[All Fields] OR PLWH[All Fields] OR PLWHA[All Fields] OR PLHIV[All Fields] OR (("persons"[MeSH Terms] OR "persons"[All Fields] OR "people"[All Fields]) AND living[All Fields] AND ("hiv"[MeSH Terms] OR "hiv"[All Fields])) OR (("persons"[MeSH Terms] OR "persons"[All Fields] OR "people"[All Fields]) AND living[All Fields] AND ("acquired immunodeficiency syndrome"[MeSH Terms] OR ("acquired"[All Fields] AND "immunodeficiency"[All Fields] AND "syndrome"[All Fields]) OR "acquired immunodeficiency syndrome"[All Fields] OR "aids"[All Fields])) OR ("hiv seropositivity"[MeSH Terms] OR ("hiv"[All Fields] AND "seropositivity"[All Fields]) OR "hiv seropositivity"[All Fields])) AND (("acute coronary syndrome"[MeSH Terms] OR ("acute"[All Fields] AND "coronary"[All Fields] AND "syndrome"[All Fields]) OR "acute coronary syndrome"[All Fields]) OR ("arteries"[MeSH Terms] OR "arteries"[All Fields] OR "arterial"[All Fields]) OR ("vascular stiffness"[MeSH Terms] OR ("vascular"[All Fields] AND "stiffness"[All Fields]) OR "vascular stiffness"[All Fields] OR ("arterial"[All Fields] AND "stiffness"[All Fields]) OR "arterial stiffness"[All Fields]) OR ("ankle brachial index"[MeSH Terms] OR ("ankle"[All Fields] AND "brachial"[All Fields] AND "index"[All Fields]) OR "ankle brachial index"[All Fields]) OR ("atherosclerosis"[MeSH Terms] OR "atherosclerosis"[All Fields]) OR Atherosclerotic[All Fields] OR (Acute[All Fields] AND ("angina pectoris"[MeSH Terms] OR ("angina"[All Fields] AND "pectoris"[All Fields]) OR "angina pectoris"[All Fields] OR "angina"[All Fields])) OR (Acute[All Fields] AND ("heart"[MeSH Terms] OR "heart"[All Fields] OR "coronary"[All Fields])) OR ("angina pectoris"[MeSH Terms] OR ("angina"[All Fields] AND "pectoris"[All Fields]) OR "angina pectoris"[All Fields] OR "angina"[All Fields]) OR ("angina pectoris"[MeSH Terms] OR ("angina"[All Fields] AND "pectoris"[All Fields]) OR "angina pectoris"[All Fields]) OR ("ischaemia"[All Fields] OR "ischemia"[MeSH Terms] OR "ischemia"[All Fields]) OR ("ischaemia"[All Fields] OR "ischemia"[MeSH Terms] OR "ischemia"[All Fields]) OR ("ischemia"[MeSH Terms] OR "ischemia"[All Fields] OR "ischemic"[All Fields]) OR ("ischemia"[MeSH Terms] OR "ischemia"[All Fields] OR "ischaemic"[All Fields]) OR ("infarction"[MeSH Terms] OR "infarction"[All Fields]) OR ("infarction"[MeSH Terms] OR "infarction"[All Fields] OR "infarct"[All Fields]) OR ("brain ischaemia"[All Fields] OR "brain ischemia"[MeSH Terms] OR ("brain"[All Fields] AND "ischemia"[All Fields]) OR "brain ischemia"[All Fields]) OR ("brain infarction"[MeSH Terms] OR ("brain"[All Fields] AND "infarction"[All Fields]) OR "brain infarction"[All Fields]) OR ("basal ganglia cerebrovascular disease"[MeSH Terms] OR ("basal"[All Fields] AND "ganglia"[All Fields] AND "cerebrovascular"[All Fields] AND "disease"[All Fields]) OR "basal ganglia cerebrovascular disease"[All Fields]) OR ("cardiovascular diseases"[MeSH Terms] OR ("cardiovascular"[All Fields] AND "diseases"[All Fields]) OR "cardiovascular diseases"[All Fields] OR ("cardiovascular"[All Fields] AND "disease"[All Fields]) OR "cardiovascular disease"[All Fields]) OR ("cardiovascular system"[MeSH Terms] OR ("cardiovascular"[All Fields] AND "system"[All Fields]) OR "cardiovascular system"[All Fields] OR "cardiovascular"[All Fields]) OR (("cardiovascular system"[MeSH Terms] OR ("cardiovascular"[All Fields] AND "system"[All Fields]) OR "cardiovascular system"[All Fields] OR "cardiovascular"[All Fields]) AND event[All Fields]) OR ("cardiovascular system"[MeSH Terms] OR ("cardiovascular"[All Fields] AND "system"[All Fields]) OR "cardiovascular system"[All Fields]) OR ("cardiovascular diseases"[MeSH Terms] OR ("cardiovascular"[All Fields] AND "diseases"[All Fields]) OR "cardiovascular diseases"[All Fields] OR ("cardiovascular"[All Fields] AND "disorder"[All Fields]) OR "cardiovascular disorder"[All Fields]) OR (("cardiovascular system"[MeSH Terms] OR ("cardiovascular"[All Fields] AND "system"[All Fields]) OR "cardiovascular system"[All Fields] OR "cardiovascular"[All Fields]) AND ("mortality"[Subheading] OR "mortality"[All Fields] OR "mortality"[MeSH Terms])) OR (("cardiovascular system"[MeSH Terms] OR ("cardiovascular"[All Fields] AND "system"[All Fields]) OR "cardiovascular system"[All Fields] OR "cardiovascular"[All Fields]) AND ("death"[MeSH Terms] OR "death"[All Fields])) OR (("cardiovascular system"[MeSH Terms] OR ("cardiovascular"[All Fields] AND "system"[All Fields]) OR "cardiovascular system"[All Fields] OR "cardiovascular"[All Fields]) AND outcome[All Fields]) OR ("cardiomyopathies"[MeSH Terms] OR "cardiomyopathies"[All Fields] OR "cardiomyopathy"[All Fields]) OR Carotid[All Fields] OR ("carotid intima-media thickness"[MeSH Terms] OR ("carotid"[All Fields] AND "intima-media"[All Fields] AND "thickness"[All Fields]) OR "carotid intima-media thickness"[All Fields] OR ("carotid"[All Fields] AND "intima"[All Fields] AND "media"[All Fields] AND "thickness"[All Fields]) OR "carotid intima media thickness"[All Fields]) OR (Carotid[All Fields] AND stiffness[All Fields]) OR ("carotid artery diseases"[MeSH Terms] OR ("carotid"[All Fields] AND "artery"[All Fields] AND "diseases"[All Fields]) OR "carotid artery diseases"[All Fields] OR ("carotid"[All Fields] AND "artery"[All Fields] AND "disease"[All Fields]) OR "carotid artery disease"[All Fields]) OR ("arteries"[MeSH Terms] OR "arteries"[All Fields] OR "artery"[All Fields]) OR ("carotid artery thrombosis"[MeSH Terms] OR ("carotid"[All Fields] AND "artery"[All Fields] AND "thrombosis"[All Fields]) OR "carotid artery thrombosis"[All Fields]) OR ("heart"[MeSH Terms] OR "heart"[All Fields] OR "cardiac"[All Fields]) OR ("heart diseases"[MeSH Terms] OR ("heart"[All Fields] AND "diseases"[All Fields]) OR "heart diseases"[All Fields] OR ("cardiac"[All Fields] AND "disease"[All Fields]) OR "cardiac disease"[All Fields]) OR ("arrhythmias, cardiac"[MeSH Terms] OR ("arrhythmias"[All Fields] AND "cardiac"[All Fields]) OR "cardiac arrhythmias"[All Fields] OR ("cardiac"[All Fields] AND "arrhythmias"[All Fields])) OR ("death"[MeSH Terms] OR "death"[All Fields] OR ("cardiac"[All Fields] AND "death"[All Fields]) OR "cardiac death"[All Fields]) OR ("heart"[MeSH Terms] OR "heart"[All Fields] OR "coronary"[All Fields]) OR (("coronary vessels"[MeSH Terms] OR ("coronary"[All Fields] AND "vessels"[All Fields]) OR "coronary vessels"[All Fields] OR ("coronary"[All Fields] AND "artery"[All Fields]) OR "coronary artery"[All Fields]) AND ("calcium"[MeSH Terms] OR "calcium"[All Fields])) OR ("coronary disease"[MeSH Terms] OR ("coronary"[All Fields] AND "disease"[All Fields]) OR "coronary disease"[All Fields] OR ("coronary"[All Fields] AND "heart"[All Fields] AND "disease"[All Fields]) OR "coronary heart disease"[All Fields]) OR ("coronary disease"[MeSH Terms] OR ("coronary"[All Fields] AND "disease"[All Fields]) OR "coronary disease"[All Fields]) OR ("coronary artery disease"[MeSH Terms] OR ("coronary"[All Fields] AND "artery"[All Fields] AND "disease"[All Fields]) OR "coronary artery disease"[All Fields]) OR ("coronary thrombosis"[MeSH Terms] OR ("coronary"[All Fields] AND "thrombosis"[All Fields]) OR "coronary thrombosis"[All Fields]) OR (("heart"[MeSH Terms] OR "heart"[All Fields] OR "coronary"[All Fields]) AND ("syndrome"[MeSH Terms] OR "syndrome"[All Fields])) OR Cerebrovascular[All Fields] OR ("cerebrovascular disorders"[MeSH Terms] OR ("cerebrovascular"[All Fields] AND "disorders"[All Fields]) OR "cerebrovascular disorders"[All Fields] OR ("cerebrovascular"[All Fields] AND "disease"[All Fields]) OR "cerebrovascular disease"[All Fields]) OR ("stroke"[MeSH Terms] OR "stroke"[All Fields] OR ("cerebrovascular"[All Fields] AND "accident"[All Fields]) OR "cerebrovascular accident"[All Fields]) OR ("cerebrovascular disorders"[MeSH Terms] OR ("cerebrovascular"[All Fields] AND "disorders"[All Fields]) OR "cerebrovascular disorders"[All Fields] OR ("cerebrovascular"[All Fields] AND "disorder"[All Fields]) OR "cerebrovascular disorder"[All Fields]) OR (("cerebral arteries"[MeSH Terms] OR ("cerebral"[All Fields] AND "arteries"[All Fields]) OR "cerebral arteries"[All Fields] OR ("cerebral"[All Fields] AND "artery"[All Fields]) OR "cerebral artery"[All Fields]) AND ("infarction"[MeSH Terms] OR "infarction"[All Fields])) OR ("cerebral arterial diseases"[MeSH Terms] OR ("cerebral"[All Fields] AND "arterial"[All Fields] AND "diseases"[All Fields]) OR "cerebral arterial diseases"[All Fields] OR ("cerebral"[All Fields] AND "arterial"[All Fields] AND "disease"[All Fields]) OR "cerebral arterial disease"[All Fields]) OR ("endothelium"[MeSH Terms] OR "endothelium"[All Fields]) OR ("endothelium"[MeSH Terms] OR "endothelium"[All Fields] OR "endothelial"[All Fields]) OR (("endothelium"[MeSH Terms] OR "endothelium"[All Fields] OR "endothelial"[All Fields]) AND ("physiopathology"[Subheading] OR "physiopathology"[All Fields] OR "dysfunction"[All Fields])) OR ("myocardium"[MeSH Terms] OR "myocardium"[All Fields] OR "myocardial"[All Fields]) OR ("myocardial ischaemia"[All Fields] OR "myocardial ischemia"[MeSH Terms] OR ("myocardial"[All Fields] AND "ischemia"[All Fields]) OR "myocardial ischemia"[All Fields] OR "coronary artery disease"[MeSH Terms] OR ("coronary"[All Fields] AND "artery"[All Fields] AND "disease"[All Fields]) OR "coronary artery disease"[All Fields] OR ("myocardial"[All Fields] AND "ischemia"[All Fields])) OR ("myocardial infarction"[MeSH Terms] OR ("myocardial"[All Fields] AND "infarction"[All Fields]) OR "myocardial infarction"[All Fields]) OR ("myocarditis"[MeSH Terms] OR "myocarditis"[All Fields]) OR ("cardiomyopathies"[MeSH Terms] OR "cardiomyopathies"[All Fields] OR "myocardiopathy"[All Fields]) OR ("myocardial infarction"[MeSH Terms] OR ("myocardial"[All Fields] AND "infarction"[All Fields]) OR "myocardial infarction"[All Fields] OR ("myocardial"[All Fields] AND "infarct"[All Fields]) OR "myocardial infarct"[All Fields]) OR (Flow[All Fields] AND mediated[All Fields] AND ("dilatation"[MeSH Terms] OR "dilatation"[All Fields] OR "dilation"[All Fields])) OR ("pulse wave analysis"[MeSH Terms] OR ("pulse"[All Fields] AND "wave"[All Fields] AND "analysis"[All Fields]) OR "pulse wave analysis"[All Fields] OR ("pulse"[All Fields] AND "wave"[All Fields] AND "velocity"[All Fields]) OR "pulse wave velocity"[All Fields]) OR ("pulse wave analysis"[MeSH Terms] OR ("pulse"[All Fields] AND "wave"[All Fields] AND "analysis"[All Fields]) OR "pulse wave analysis"[All Fields]) OR ("ischaemic heart disease"[All Fields] OR "myocardial ischemia"[MeSH Terms] OR ("myocardial"[All Fields] AND "ischemia"[All Fields]) OR "myocardial ischemia"[All Fields] OR ("ischemic"[All Fields] AND "heart"[All Fields] AND "disease"[All Fields]) OR "ischemic heart disease"[All Fields] OR "coronary artery disease"[MeSH Terms] OR ("coronary"[All Fields] AND "artery"[All Fields] AND "disease"[All Fields]) OR "coronary artery disease"[All Fields] OR ("ischemic"[All Fields] AND "heart"[All Fields] AND "disease"[All Fields])) OR ("heart"[MeSH Terms] OR "heart"[All Fields]) OR ("heart diseases"[MeSH Terms] OR ("heart"[All Fields] AND "diseases"[All Fields]) OR "heart diseases"[All Fields] OR ("heart"[All Fields] AND "disease"[All Fields]) OR "heart disease"[All Fields]) OR ("myocardial infarction"[MeSH Terms] OR ("myocardial"[All Fields] AND "infarction"[All Fields]) OR "myocardial infarction"[All Fields] OR ("heart"[All Fields] AND "attack"[All Fields]) OR "heart attack"[All Fields]) OR ("heart failure"[MeSH Terms] OR ("heart"[All Fields] AND "failure"[All Fields]) OR "heart failure"[All Fields]) OR (("heart"[MeSH Terms] OR "heart"[All Fields]) AND ("infarction"[MeSH Terms] OR "infarction"[All Fields] OR "infarct"[All Fields])) OR Hypoxia-ischemia[All Fields] OR Hypoxic-ischemic[All Fields] OR ("carotid artery, internal, dissection"[MeSH Terms] OR ("carotid"[All Fields] AND "artery"[All Fields] AND "internal"[All Fields] AND "dissection"[All Fields]) OR ("internal"[All Fields] AND "carotid"[All Fields] AND "artery"[All Fields] AND "dissection"[All Fields]) OR "internal carotid artery dissection"[All Fields]) OR ("intracranial embolism and thrombosis"[MeSH Terms] OR ("intracranial"[All Fields] AND "embolism"[All Fields] AND "thrombosis"[All Fields]) OR "intracranial embolism and thrombosis"[All Fields]) OR ("stroke"[MeSH Terms] OR "stroke"[All Fields]) OR (Subclinical[All Fields] AND ("atherosclerosis"[MeSH Terms] OR "atherosclerosis"[All Fields])) OR ("blood vessels"[MeSH Terms] OR ("blood"[All Fields] AND "vessels"[All Fields]) OR "blood vessels"[All Fields] OR "vascular"[All Fields]) OR ("vascular diseases"[MeSH Terms] OR ("vascular"[All Fields] AND "diseases"[All Fields]) OR "vascular diseases"[All Fields] OR ("vascular"[All Fields] AND "disease"[All Fields]) OR "vascular disease"[All Fields]) OR ("vertebral artery dissection"[MeSH Terms] OR ("vertebral"[All Fields] AND "artery"[All Fields] AND "dissection"[All Fields]) OR "vertebral artery dissection"[All Fields]) OR ("angina, unstable"[MeSH Terms] OR ("angina"[All Fields] AND "unstable"[All Fields]) OR "unstable angina"[All Fields] OR ("unstable"[All Fields] AND "angina"[All Fields])) OR (Unstable[All Fields] AND ("heart"[MeSH Terms] OR "heart"[All Fields] OR "coronary"[All Fields])) OR ("peripheral arterial disease"[MeSH Terms] OR ("peripheral"[All Fields] AND "arterial"[All Fields] AND "disease"[All Fields]) OR "peripheral arterial disease"[All Fields] OR ("peripheral"[All Fields] AND "artery"[All Fields] AND "disease"[All Fields]) OR "peripheral artery disease"[All Fields]) OR ("peripheral arterial disease"[MeSH Terms] OR ("peripheral"[All Fields] AND "arterial"[All Fields] AND "disease"[All Fields]) OR "peripheral arterial disease"[All Fields]) OR ("peripheral vascular diseases"[MeSH Terms] OR ("peripheral"[All Fields] AND "vascular"[All Fields] AND "diseases"[All Fields]) OR "peripheral vascular diseases"[All Fields] OR ("peripheral"[All Fields] AND "vascular"[All Fields] AND "disease"[All Fields]) OR "peripheral vascular disease"[All Fields]) OR (("blood vessels"[MeSH Terms] OR ("blood"[All Fields] AND "vessels"[All Fields]) OR "blood vessels"[All Fields] OR "vascular"[All Fields]) AND ("plaque, amyloid"[MeSH Terms] OR ("plaque"[All Fields] AND "amyloid"[All Fields]) OR "amyloid plaque"[All Fields] OR "plaque"[All Fields] OR "dental plaque"[MeSH Terms] OR ("dental"[All Fields] AND "plaque"[All Fields]) OR "dental plaque"[All Fields])) OR "ACS"[All Fields] OR "AMI"[All Fields] OR "CVD"[All Fields] OR "MI"[All Fields] OR "CHD"[All Fields] OR "CIMT"[All Fields] OR "FMD"[All Fields] OR "PWV"[All Fields] OR "CAC"[All Fields] OR "ABI"[All Fields]) AND (("thailand"[MeSH Terms] OR "thailand"[All Fields]) OR ("cambodia"[MeSH Terms] OR "cambodia"[All Fields]) OR ("myanmar"[MeSH Terms] OR "myanmar"[All Fields]) OR ("myanmar"[MeSH Terms] OR "myanmar"[All Fields] OR "burma"[All Fields]) OR ("bhutan"[MeSH Terms] OR "bhutan"[All Fields]) OR ("papua new guinea"[MeSH Terms] OR ("papua"[All Fields] AND "new"[All Fields] AND "guinea"[All Fields]) OR "papua new guinea"[All Fields]) OR ("melanesia"[MeSH Terms] OR "melanesia"[All Fields] OR ("solomon"[All Fields] AND "islands"[All Fields]) OR "solomon islands"[All Fields]) OR ("malaysia"[MeSH Terms] OR "malaysia"[All Fields]) OR ("indonesia"[MeSH Terms] OR "indonesia"[All Fields]) OR ("asian continental ancestry group"[MeSH Terms] OR ("asian"[All Fields] AND "continental"[All Fields] AND "ancestry"[All Fields] AND "group"[All Fields]) OR "asian continental ancestry group"[All Fields] OR "thai"[All Fields]) OR ("asian continental ancestry group"[MeSH Terms] OR ("asian"[All Fields] AND "continental"[All Fields] AND "ancestry"[All Fields] AND "group"[All Fields]) OR "asian continental ancestry group"[All Fields] OR "cambodian"[All Fields]) OR ("asian continental ancestry group"[MeSH Terms] OR ("asian"[All Fields] AND "continental"[All Fields] AND "ancestry"[All Fields] AND "group"[All Fields]) OR "asian continental ancestry group"[All Fields] OR "burmese"[All Fields]) OR Bhutanese[All Fields] OR (Papua[All Fields] AND New[All Fields] AND Guinean[All Fields]) OR (Solomon[All Fields] AND islander[All Fields]) OR Malaysian[All Fields] OR Indonesian[All Fields])

*Embase - [All texts] - All times*

*572 articles*

('hiv'/exp OR hiv OR 'human immunodeficiency virus'/exp OR 'human immunodeficiency virus' OR (('human'/exp OR human) AND ('immunodeficiency'/exp OR immunodeficiency) AND ('virus'/exp OR virus)) OR 'human immune deficiency virus' OR (('human'/exp OR human) AND ('immune'/exp OR immune) AND ('deficiency'/exp OR deficiency) AND ('virus'/exp OR virus)) OR 'aids'/exp OR aids OR 'acquired immunodeficiency syndrome'/exp OR 'acquired immunodeficiency syndrome' OR (acquired AND ('immunodeficiency'/exp OR immunodeficiency) AND ('syndrome'/exp OR syndrome)) OR 'acquired immune deficiency syndrome'/exp OR 'acquired immune deficiency syndrome' OR (acquired AND ('immune'/exp OR immune) AND ('deficiency'/exp OR deficiency) AND ('syndrome'/exp OR syndrome)) OR 'hiv positive' OR (('hiv'/exp OR hiv) AND positive) OR 'hiv positive patients' OR (('hiv'/exp OR hiv) AND positive AND ('patients'/exp OR patients)) OR 'human immunodeficiency virus infection'/exp OR 'human immunodeficiency virus infection' OR (('human'/exp OR human) AND ('immunodeficiency'/exp OR immunodeficiency) AND ('virus'/exp OR virus) AND ('infection'/exp OR infection)) OR 'hiv 1'/exp OR 'hiv 1' OR 'hiv 2'/exp OR 'hiv 2' OR 'human immuno-deficiency virus'/exp OR 'human immuno-deficiency virus' OR (('human'/exp OR human) AND 'immuno deficiency' AND ('virus'/exp OR virus)) OR 'hiv infections'/exp OR 'hiv infections' OR (('hiv'/exp OR hiv) AND ('infections'/exp OR infections)) OR 'hiv infected' OR plwh OR plwha OR plhiv OR 'people living with hiv'/exp OR 'people living with hiv' OR (people AND ('living'/exp OR living) AND with AND ('hiv'/exp OR hiv)) OR 'people living with aids' OR (people AND ('living'/exp OR living) AND with AND ('aids'/exp OR aids)) OR 'hiv seropositivity'/exp OR 'hiv seropositivity' OR (('hiv'/exp OR hiv) AND ('seropositivity'/exp OR seropositivity))) AND (('acute coronary syndrome'/exp OR 'acute coronary syndrome' OR (acute AND coronary AND ('syndrome'/exp OR syndrome)) OR arterial OR 'arterial stiffness'/exp OR 'arterial stiffness' OR (arterial AND ('stiffness'/exp OR stiffness)) OR 'ankle brachial index'/exp OR 'ankle brachial index' OR (('ankle'/exp OR ankle) AND brachial AND ('index'/exp OR index)) OR 'atherosclerosis'/exp OR atherosclerosis OR atherosclerotic OR 'acute angina' OR (acute AND ('angina'/exp OR angina)) OR 'acute coronary' OR (acute AND coronary) OR 'angina'/exp OR angina OR 'angina pectoris'/exp OR 'angina pectoris' OR (('angina'/exp OR angina) AND pectoris) OR 'ischemia'/exp OR ischemia OR 'ischaemia'/exp OR ischaemia OR ischemic OR ischaemic OR 'infarction'/exp OR infarction OR 'infarct'/exp OR infarct OR 'brain ischemia'/exp OR 'brain ischemia' OR (('brain'/exp OR brain) AND ('ischemia'/exp OR ischemia)) OR 'brain infarction'/exp OR 'brain infarction' OR (('brain'/exp OR brain) AND ('infarction'/exp OR infarction)) OR 'basal ganglia cerebrovascular disease'/exp OR 'basal ganglia cerebrovascular disease' OR (basal AND ('ganglia'/exp OR ganglia) AND cerebrovascular AND ('disease'/exp OR disease)) OR 'cardiovascular disease'/exp OR 'cardiovascular disease' OR (('cardiovascular'/exp OR cardiovascular) AND ('disease'/exp OR disease)) OR 'cardiovascular'/exp OR cardiovascular OR 'cardiovascular event'/exp OR 'cardiovascular event' OR (('cardiovascular'/exp OR cardiovascular) AND event) OR 'cardiovascular system'/exp OR 'cardiovascular system' OR (('cardiovascular'/exp OR cardiovascular) AND system) OR 'cardiovascular disorder'/exp OR 'cardiovascular disorder' OR (('cardiovascular'/exp OR cardiovascular) AND ('disorder'/exp OR disorder)) OR 'cardiovascular mortality'/exp OR 'cardiovascular mortality' OR (('cardiovascular'/exp OR cardiovascular) AND ('mortality'/exp OR mortality)) OR 'cardiovascular death'/exp OR 'cardiovascular death' OR (('cardiovascular'/exp OR cardiovascular) AND ('death'/exp OR death)) OR 'cardiovascular outcome'/exp OR 'cardiovascular outcome' OR (('cardiovascular'/exp OR cardiovascular) AND ('outcome'/exp OR outcome)) OR 'cardiomyopathy'/exp OR cardiomyopathy OR 'carotid'/exp OR carotid OR 'carotid intima-media thickness'/exp OR 'carotid intima-media thickness' OR (('carotid'/exp OR carotid) AND 'intima media' AND ('thickness'/exp OR thickness)) OR 'carotid stiffness'/exp OR 'carotid stiffness' OR (('carotid'/exp OR carotid) AND ('stiffness'/exp OR stiffness)) OR 'carotid artery disease'/exp OR 'carotid artery disease' OR (('carotid'/exp OR carotid) AND ('artery'/exp OR artery) AND ('disease'/exp OR disease)) OR 'artery'/exp OR artery OR 'carotid artery thrombosis'/exp OR 'carotid artery thrombosis' OR (('carotid'/exp OR carotid) AND ('artery'/exp OR artery) AND ('thrombosis'/exp OR thrombosis)) OR cardiac OR 'cardiac disease'/exp OR 'cardiac disease' OR (cardiac AND ('disease'/exp OR disease)) OR 'cardiac arrhythmias' OR (cardiac AND arrhythmias) OR 'cardiac death'/exp OR 'cardiac death' OR (cardiac AND ('death'/exp OR death)) OR coronary OR 'coronary artery calcium'/exp OR 'coronary artery calcium' OR (coronary AND ('artery'/exp OR artery) AND ('calcium'/exp OR calcium)) OR 'coronary heart disease'/exp OR 'coronary heart disease' OR (coronary AND ('heart'/exp OR heart) AND ('disease'/exp OR disease)) OR 'coronary disease'/exp OR 'coronary disease' OR (coronary AND ('disease'/exp OR disease)) OR 'coronary artery disease'/exp OR 'coronary artery disease' OR (coronary AND ('artery'/exp OR artery) AND ('disease'/exp OR disease)) OR 'coronary thrombosis'/exp OR 'coronary thrombosis' OR (coronary AND ('thrombosis'/exp OR thrombosis)) OR 'coronary syndrome'/exp OR 'coronary syndrome' OR (coronary AND ('syndrome'/exp OR syndrome)) OR cerebrovascular OR 'cerebrovascular disease'/exp OR 'cerebrovascular disease' OR (cerebrovascular AND ('disease'/exp OR disease)) OR 'cerebrovascular accident'/exp OR 'cerebrovascular accident' OR (cerebrovascular AND ('accident'/exp OR accident)) OR 'cerebrovascular disorder'/exp OR 'cerebrovascular disorder' OR (cerebrovascular AND ('disorder'/exp OR disorder)) OR 'cerebral artery infarction' OR (cerebral AND ('artery'/exp OR artery) AND ('infarction'/exp OR infarction)) OR 'cerebral arterial disease' OR (cerebral AND arterial AND ('disease'/exp OR disease)) OR 'endothelium'/exp OR endothelium OR endothelial OR 'endothelial dysfunction'/exp OR 'endothelial dysfunction' OR (endothelial AND dysfunction) OR myocardial OR 'myocardial ischemia'/exp OR 'myocardial ischemia' OR (myocardial AND ('ischemia'/exp OR ischemia)) OR 'myocardial infarction'/exp OR 'myocardial infarction' OR (myocardial AND ('infarction'/exp OR infarction)) OR 'myocarditis'/exp OR myocarditis OR 'myocardiopathy'/exp OR myocardiopathy OR 'myocardial infarct'/exp OR 'myocardial infarct' OR (myocardial AND ('infarct'/exp OR infarct)) OR 'flow mediated dilation'/exp OR 'flow mediated dilation' OR (('flow'/exp OR flow) AND mediated AND dilation) OR 'pulse wave velocity'/exp OR 'pulse wave velocity' OR (('pulse'/exp OR pulse) AND ('wave'/exp OR wave) AND ('velocity'/exp OR velocity)) OR 'pulse wave analysis'/exp OR 'pulse wave analysis' OR (('pulse'/exp OR pulse) AND ('wave'/exp OR wave) AND ('analysis'/exp OR analysis)) OR 'ischemic heart disease'/exp OR 'ischemic heart disease' OR (ischemic AND ('heart'/exp OR heart) AND ('disease'/exp OR disease)) OR 'heart'/exp OR heart OR 'heart disease'/exp OR 'heart disease' OR (('heart'/exp OR heart) AND ('disease'/exp OR disease)) OR 'heart attack'/exp OR 'heart attack' OR (('heart'/exp OR heart) AND attack) OR 'heart failure'/exp OR 'heart failure' OR (('heart'/exp OR heart) AND ('failure'/exp OR failure)) OR 'heart infarct'/exp OR 'heart infarct' OR (('heart'/exp OR heart) AND ('infarct'/exp OR infarct)) OR 'hypoxia ischemia' OR 'hypoxic ischemic' OR 'internal carotid artery dissection'/exp OR 'internal carotid artery dissection' OR (internal AND ('carotid'/exp OR carotid) AND ('artery'/exp OR artery) AND ('dissection'/exp OR dissection)) OR 'intracranial embolism'/exp OR 'intracranial embolism' OR (intracranial AND ('embolism'/exp OR embolism))) AND ('thrombosis'/exp OR thrombosis) OR 'stroke'/exp OR stroke OR 'subclinical atherosclerosis'/exp OR 'subclinical atherosclerosis' OR (subclinical AND ('atherosclerosis'/exp OR atherosclerosis)) OR vascular OR 'vascular disease'/exp OR 'vascular disease' OR (vascular AND ('disease'/exp OR disease)) OR 'vertebral artery dissection'/exp OR 'vertebral artery dissection' OR (vertebral AND ('artery'/exp OR artery) AND ('dissection'/exp OR dissection)) OR 'unstable angina'/exp OR 'unstable angina' OR (unstable AND ('angina'/exp OR angina)) OR 'unstable coronary' OR (unstable AND coronary) OR 'peripheral artery disease'/exp OR 'peripheral artery disease' OR (peripheral AND ('artery'/exp OR artery) AND ('disease'/exp OR disease)) OR 'peripheral arterial disease'/exp OR 'peripheral arterial disease' OR (peripheral AND arterial AND ('disease'/exp OR disease)) OR 'peripheral vascular disease'/exp OR 'peripheral vascular disease' OR (peripheral AND vascular AND ('disease'/exp OR disease)) OR 'vascular plaque' OR (vascular AND ('plaque'/exp OR plaque)) OR 'acs' OR 'ami' OR 'cvd' OR 'mi' OR 'chd' OR 'cimt' OR 'fmd' OR 'pwv' OR 'cac' OR 'abi') AND ('thailand'/exp OR thailand OR 'cambodia'/exp OR cambodia OR 'myanmar'/exp OR myanmar OR 'burma'/exp OR burma OR 'bhutan'/exp OR bhutan OR 'papua new guinea'/exp OR 'papua new guinea' OR (('papua'/exp OR papua) AND new AND ('guinea'/exp OR guinea)) OR 'solomon islands'/exp OR 'solomon islands' OR (solomon AND ('islands'/exp OR islands)) OR 'malaysia'/exp OR malaysia OR 'indonesia'/exp OR indonesia OR 'thai'/exp OR thai OR 'cambodian'/exp OR cambodian OR 'burmese'/exp OR burmese OR 'bhutanese'/exp OR bhutanese OR 'papua new guinean'/exp OR 'papua new guinean' OR (('papua'/exp OR papua) AND new AND ('guinean'/exp OR guinean)) OR 'solomon islander' OR (solomon AND islander) OR 'malaysian'/exp OR malaysian OR 'indonesian'/exp OR indonesian)

*The Cochrane Database of Systematic review- [All texts] - All times*

*334 Reviews*

324 Cochrane Reviews matching (HIV OR Human immunodeficiency virus OR Human immune deficiency virus OR AIDS OR Acquired immunodeficiency syndrome OR Acquired immune deficiency syndrome OR HIV positive OR HIV positive patients OR Human immunodeficiency virus infection OR HIV-1 OR HIV-2 OR Human immuno-deficiency virus OR HIV infections OR HIV-infected OR PLWH OR PLWHA OR PLHIV OR People living with HIV OR People living with AIDS OR HIV seropositivity) AND (Acute coronary syndrome OR Arterial OR Arterial stiffness OR Ankle brachial index OR Atherosclerosis OR Atherosclerotic OR Acute angina OR Acute coronary OR Angina OR Angina pectoris OR Ischemia OR Ischaemia OR Ischemic OR Ischaemic OR Infarction OR Infarct OR Brain ischemia OR Brain infarction OR Basal ganglia cerebrovascular disease OR Cardiovascular disease OR Cardiovascular OR Cardiovascular event OR Cardiovascular system OR Cardiovascular disorder OR Cardiovascular mortality OR Cardiovascular death OR Cardiovascular outcome OR Cardiomyopathy OR Carotid OR Carotid intima-media thickness OR Carotid stiffness OR Carotid artery disease OR Artery OR Carotid artery thrombosis OR Cardiac OR Cardiac disease OR Cardiac arrhythmias OR Cardiac death OR Coronary OR Coronary artery calcium OR Coronary heart disease OR Coronary disease OR Coronary artery disease OR Coronary thrombosis OR Coronary syndrome OR Cerebrovascular OR Cerebrovascular disease OR Cerebrovascular accident OR Cerebrovascular disorder OR Cerebral artery infarction OR Cerebral arterial disease OR Endothelium OR Endothelial OR Endothelial dysfunction OR Myocardial OR Myocardial ischemia OR Myocardial infarction OR Myocarditis OR Myocardiopathy OR Myocardial infarct OR Flow mediated dilation OR Pulse wave velocity OR Pulse wave analysis OR Ischemic heart disease OR Heart OR Heart disease OR Heart attack OR Heart failure OR Heart infarct OR Hypoxia-ischemia OR Hypoxic-ischemic OR Internal carotid artery dissection OR Intracranial embolism and thrombosis OR Stroke OR Subclinical atherosclerosis OR Vascular OR Vascular disease OR Vertebral artery dissection OR Unstable angina OR Unstable coronary OR Peripheral artery disease OR Peripheral arterial disease OR Peripheral vascular disease OR Vascular plaque OR “ACS” OR “AMI” OR “CVD” OR “MI” OR “CHD” OR “CIMT” OR “FMD” OR “PWV” OR “CAC” OR “ABI”) AND (Thailand OR Cambodia OR Myanmar OR Burma OR Bhutan OR Papua New Guinea OR Solomon Islands OR Malaysia OR Indonesia OR Thai OR Cambodian OR Burmese OR Bhutanese OR Papua New Guinean OR Solomon islander OR Malaysian OR Indonesian) in All Text - (Word variations have been searched)

----------------------------------------------------------------------------------------------------------------
