# Supplementary material for: Prevalence and risk factors of cardiovascular disease among people living with HIV in the Asia-Pacific region: a systematic review
Source: BMC Public Health. 2023 Mar 13;23:477. doi: 10.1186/s12889-023-15321-7 (PMC10009940; doi:10.1186/s12889-023-15321-7)
Supplement: Supplementary file 2 — Supplementary Material 2 [file 12889_2023_15321_MOESM2_ESM.docx]

**Additional File 2**

**Table 1 Quality assessment of the included cross-sectional studies using the Newcastle-Ottawa scale**

|  | **Question** | **1** | **2** | **3** | **4** | **5** | **6** | **7** |
| --- | --- | --- | --- | --- | --- | --- | --- | --- |
| ***Selection*** | | | | | | | | |
| 1 | **Representativeness of the sample** | | | | | | | |
|  | 1. Truly representative of the average in the target population. (all subjects or random sampling) |  |  |  |  |  | ***** |  |
|  | 1. Somewhat representative of the average in the target population. (non-random sampling) | ***** | ***** | ***** | ***** | ***** |  | ***** |
|  | 1. Selected group of users or convenience sample   e.g., volunteers, members, nurses |  |  |  |  |  |  |  |
|  | 1. No description of the sampling strategy |  |  |  |  |  |  |  |
| 2 | **Sample size** | | | | | | | |
|  | 1. Justified and satisfactory. |  |  | * |  |  |  |  |
|  | 1. Not justified. | * | * |  | * | * | * | * |
| 3 | **Non-respondents** |  |  |  |  |  |  |  |
|  | 1. Comparability between respondents and non-respondents characteristics is established, and the response rate is satisfactory. |  |  |  |  |  |  |  |
|  | 1. The response rate is unsatisfactory, or the comparability between respondents and non-respondents is unsatisfactory. |  |  |  |  |  |  |  |
|  | 1. No description of the response rate or the characteristics of the responders and the non-responders. | ***** | ***** | ***** | ***** | ***** | ***** | ***** |
|  | **Ascertainment of exposure (risk factor)** |  |  |  |  |  |  |  |
|  | Validated measurement tool. | ***** | ***** | ***** | ***** | ***** | ***** | ***** |
|  | Non-validated measurement tool, but the tool is available or described. |  |  |  |  |  |  |  |
|  | No description of the measurement tool. |  |  |  |  |  |  |  |
|  | ***Comparability*** |  |  |  |  |  |  |  |
| 5 | **The subjects in different outcome groups are comparable, based on the study design or analysis. Confounding factors are controlled.** |  |  |  |  |  |  |  |
|  | The study controls for the most important factor (select one). |  |  |  |  |  |  |  |
|  | The study control for any additional factor. | ***** | ***** | ***** | ***** | ***** | ***** | ***** |
|  | **Assessment of outcome** |  |  |  |  |  |  |  |
|  | Independent or blind assessment | ***** | ***** | ***** | ***** | ***** | ***** | ***** |
|  | Record linkage |  |  |  |  |  |  |  |
|  | Self-report |  |  |  |  |  |  |  |
|  | No description |  |  |  |  |  |  |  |
|  | **Assessment of outcome** |  |  |  |  |  |  |  |
|  | Independent or blind assessment | ***** | ***** | ***** | ***** | ***** | ***** | ***** |
|  | Record linkage |  |  |  |  |  |  |  |
|  | Self-report |  |  |  |  |  |  |  |
|  | No description |  |  |  |  |  |  |  |
|  | **Statistical test** |  |  |  |  |  |  |  |
|  | The statistical test used to analyze the data is clearly described and appropriate, and the measurement of the association is presented, including confidence intervals and the probability level (p-value). | ***** | ***** | ***** | ***** | ***** | ***** | ***** |
|  | The statistical test is not appropriate, not described or incomplete. |  |  |  |  |  |  |  |
|  | AHQR standards | G | G | G | G | G | G | G |

**Table 1**

1. Aurpibul et al.^(22)^, 2. Putcharoen et al.^(23)^, 3. Siwamogsatham et al.^(24)^, 4. Utama et al.^(25)^, 5. Rajasuriar et al.^(26)^6. Aurpibul et al.^(27)^, 7. Nakaranurack, et al.^(28)^

* = Quality met, G= Good quality

Table 2 Quality assessment of the included cohort studies using the Newcastle-Ottawa scale

|  | **Question** | **1** | **2** | **3** |
| --- | --- | --- | --- | --- |
| ***Selection*** | | | | |
| 1 | **Representativeness of the exposed cohort** | | | |
|  | 1. truly representative of the average in the target population in the community |  | ***** | ***** |
|  | 1. somewhat representative of the average in the target population in the community | ***** |  |  |
|  | 1. selected group of users e.g., nurses, volunteers |  |  |  |
|  | 1. no description of the derivation of the cohort |  |  |  |
| 2 | **Selection of the non-exposed cohort** | | | |
|  | 1. drawn from the same community as the exposed cohort | ***** | ***** | ***** |
|  | 1. drawn from a different source |  |  |  |
|  | 1. no description of the derivation of the non-exposed cohort |  |  |  |
| 3 | **Ascertainment of exposure** | | | |
|  | 1. secure record (e.g., surgical records) | ***** | ***** | ***** |
|  | 1. structured interview |  |  |  |
| 4 | **Demonstration that outcome of interest was not present at start of study** | | | |
|  | 1. yes |  | ***** | ***** |
|  | 1. no | ***** |  |  |
| ***Comparability*** | | | | |
| 5 | **Comparability of cohorts on the basis of the design or analysis** | | | |
|  | 1. study controls for _____________ (select the most important factor) | **-** | **-** | **-** |
|  | 1. study controls for any additional factor (These criteria could be modified to indicate specific control for a second important factor.) | ***** | **-** | **-** |
| 6 | **Assessment of outcome** | | | |
|  | 1. independent blind assessment |  | ***** | ***** |
|  | 1. record linkage | ***** |  |  |
|  | 1. self-report |  |  |  |
|  | 1. no description |  |  |  |
| 7 | **Was follow-up long enough for outcomes to occur** | | | |
|  | 1. yes (select an adequate follow up period for outcome of interest) | ***** | ***** | ***** |
|  | 1. no |  |  |  |
| 8 | **Adequacy of follow-up of cohorts** | | | |
|  | 1. complete follow up - all subjects accounted for |  |  | ***** |
|  | 1. subjects lost to follow up unlikely to introduce bias - small number lost > (select an adequate %) follow up, or description provided of those lost) | *** (LTF=1.8%)** |  |  |
|  | 1. follow up rate % (select an adequate %) and no description of those lost |  | *****  **(LTF=17%)** |  |
|  | 1. no statement |  |  |  |
|  | **AHQR standards** | **G** | **P** | **P** |

1. Sitticharoenchai et al.^(21)^, 2. Karim et al.^(19)^, 3. Subsai et al.^(20)^

* = Quality met, G=Good quality, P=Poor quality, LTF = loss-to-follow up

**Table 3 Quality assessment of the included case-control study using the Newcastle-Ottawa scale**

|  | **Question** | **Lee et al. 2012^(29)^** |
| --- | --- | --- |
| ***Selection*** | | |
| 1 | **Is the case definition adequate?** | |
|  | 1. yes, with independent validation | ***** |
|  | 1. yes, e.g., record linkage or based on self-reports |  |
|  | 1. no description |  |
| 2 | **Representativeness of the cases** | |
|  | 1. consecutive or obviously representative series of cases | ***** |
|  | 1. potential for selection biases or not stated |  |
| 3 | **Selection of Controls** | |
|  | 1. community controls |  |
|  | 1. hospital controls | ***** |
|  | 1. no description |  |
| 4 | **Definition of Controls** | |
|  | 1. no history of diseases (endpoint) | ***** |
|  | 1. no description of sources |  |
| ***Comparability*** | | |
| 5 | **Comparability of cases and controls on the basis of the design or analysis** | |
|  | 1. study controls for (Select the most important factor.) |  |
|  | 1. study controls for any additional factor (These criteria could be modified to indicate specific control for a second important factor.) | ***** |
| ***Exposure*** | | |
| 6 | **Ascertainment of exposure** | |
|  | 1. secure record (e.g., surgical records) | ***** |
|  | 1. structured interview where blind to case-control status |  |
|  | 1. interview not blinded to case-control status |  |
|  | 1. written self-report or medical record only |  |
|  | 1. no description |  |
| 7 | **Same method of ascertainment for cases and controls** | |
|  | 1. yes | ***** |
|  | 1. no |  |
| 8 | **Non-Response rate** | |
|  | 1. same rate for both groups |  |
|  | 1. non respondents described | ***** |
|  | 1. rate different and no designation |  |
|  | **AHQR standards** | **G** |

* = Quality met, G=good quality
